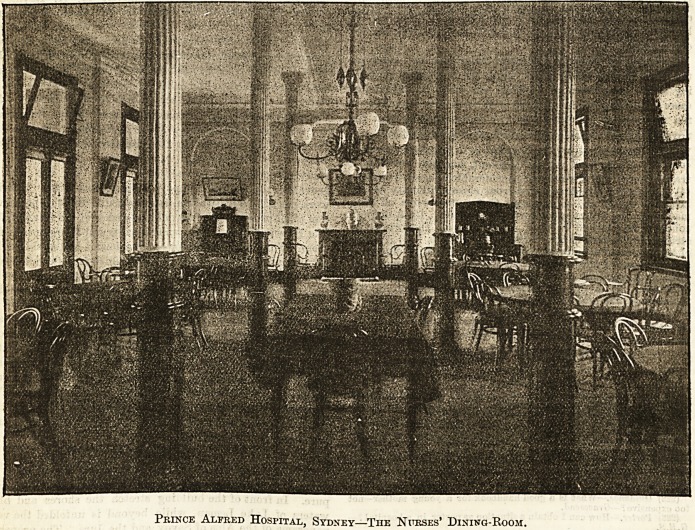# Extra Supplement.—The Nursing Mirror

**Published:** 1893-11-18

**Authors:** 


### "The Hospital" Nursing Mirror.

Being the Extra Nursing Supplement of "The Hospital" Newspaper.

[Contributions for this Supplement should be addressed to the Editor, The Hospital, 428, Strand, London, W.C., and should have the word "Nursing" plainly written in left-hand top corner of the envelope.]

### News from the Nursing World.

### OUR CHRISTMAS COMPETITIONS.

Each year our Christmas parcels are so warmly welcomed and appreciated by the sick poor in hospital wards that we trust every successive season will bring a steady increase in the number of garments provided for this annual distribution. The prizes will be: (1) For the best pair of socks knitted by a nurse, 5s.; (2) for the best pair knitted by any Hospital reader, 5s.; (3) for the best made flannel shirt, 10s.; (4) for the best flannel or flannelette bed-jacket, 10s.; (5) for the best flannel petticoat, 10s.; (6) for the best made and simplest shaped dressing gown made and cut out by a nurse, 20s. Long seams may be done by machine. All parcels must reach The Hospital Office, 428, Strand, in the first week of December, and they should bear the words "Needlework Competition" in left-hand corner of address label, and also the name of the sender.

### A SEARCHING INVESTIGATION IMPERATIVE!

At the Manchester Winter Assizes, before a special jury, Nurse Lydia A. Fincken has recently successfully established her right to submit herself for examination with a view of obtaining her certificate of competency promised by the regulations at the expiration of her three years' training. She is said to have discharged her duties satisfactorily during the three years, but failed to please the Matron, who sent her to nurse small-pox cases under conditions so shocking as to cause the Matron to refuse to supply more nurses, although they were badly wanted. We cannot give a decided opinion on the merits of the opposing views of the Matron and Nurse Fincken as to the small-pox incident without fuller information than we at present possess. The statements made by counsel at the trial proved, however, that the reputation of the Royal Infirmary, Manchester, demands a searching inquiry into the whole case by a small Special Committee charged with the duty of taking evidence on oath, the proceedings at which should be public. If the hospitals are to continue to supply nurses to private cases they must convince the public that they are prepared to take efficient steps to protect their nurses from abuses and conditions such as those Nurse Fincken had to face without counsel or aid from her chiefs. This case puts both the Matron and the Nurse on trial, and cannot be permitted to remain where it stands at present after the proceedings in Court.

### UNIQUE TESTIMONIALS.

It is to be hoped that the Islington Guardians are singular in their views about testimonials. They are accustomed apparently to bestow these upon women who have nursed in their infirmary, without consultation either with medical officer or matron. The recent inquiry brought forth grievous revelations as to dishonesty, impertinence, and immorality on the part of various nurses who had been in the employ of the guardians; and the doctor, during the eleven years he has held office at the infirmary, has only been asked on some four occasions by the board to give his opinion as to the work and character of these nurses. The matron's position has been most painful; whilst one nurse "slanged" her in the ward; of another, to whom the board had given a good testimonial, she reported, "she constantly disobeyed my orders, and went out one night and did not return." The guardians have failed to realise the nature of their responsibilities towards the sick poor, as well as the obligations to respect and co-operate with the doctor and matron appointed by themselves. There being only fifteen day nurses to six hundred patients, somewhat explains the remark of the matron, "I have for a very long time put up with things in the infirmary which other matrons would not have tolerated, because I think our nurses are over-worked, and have not comfortable quarters."

### SPECIAL TRAINING.

There is good reason to hope that in the next generation "attendants on the insane" will be superseded by nurses specially qualified to take charge of patients with brain disease. Excellent facilities are now given for the training of probationers in mental nursing, and ere long the value of these opportunities will doubtless be more fully appreciated. Being only one branch of trained nursing it must of course be supplemented by other experience. However good the instruction given in asylums the practical work has limitations. Therefore to become an all-round good nurse a woman needs the knowledge only to be gained in a general hospital or an infirmary, where the matron is herself a trained nurse. The certificate gained in an asylum adds greatly to the value of a nurse, particularly in private work when it obtains for her higher fees than she would otherwise receive. It is by nurses who have already trained in the care of sane folks that asylum experience is most valued. If, however, they prefer to begin with the study of mental nursing they must not be content to end there, as it is obvious that employment in other branches will not be obtainable unless knowledge of the sane be added to experience in the care of those "mentally afflicted."

### WANTED—TRAINED NURSES.

Coroners pointedly call attention to the urgent need for the employment of trained nurses in workhouse infirmaries, and certainly their testimony should not be disregarded. It is powerfully supported by the facts which the *British Medical Journal* published last week with figures, giving the proportion of patients to attendants. Whilst trained nurses are advocated by these authorities, the Leicester Guardians have contented themselves with appointing a person whose preparation for this responsible post consists apparently in "the qualification of a St. John Ambulance certificate and a medal." It is to be hoped that this so-called "qualification" is an addition and not a substitute for adequate training and experience in the management of the sick and infirm poor. Another infirmary advertises for candidates who can write well. If Guardians have no larger and clearer views than these as to the qualifications needed in nurses, they had better be at the pains to ask advice from those better informed than themselves.

### "BOYS WILL BE BOYS."

Even old bachelors and prim maiden aunts condone the misdemeanours of their nephews, because "boys will be boys" they cheerfully declare. In other words, boys must be allowed to make plenty of noise, and otherwise to conduct themselves like the high-spirited young animals they are. The sooner the managers of fever hospitals recognise this fact the better for their adult patients. Little schoolboys soon recover their spirits and seldom lose their voices during the attack of scarlet fever which cuts them off from their natural diversions. Happily they easily adapt themselves to altered circumstances, and are contented enough if they have reasonable license with regard to noise and nonsense. But when new and acute cases are admitted into the same ward with lively youngsters, assuredly the poor men deserve pity. It isn't fair on the boys to "hush them up" incessantly during their six weeks' residence, and most certainly it is not fair on the men to subject them to constant disturbances. A boys' ward would be a reasonable and a highly appreciated addition to every hospital set apart for the nursing of infectious diseases.

### PRUDENT PRECAUTIONS.

"Women first act foolishly, and then come to us for advice to get them out of their difficulties," said a man who had just heard a woeful tale of disastrous investments from an impulsive and rather impecunious female relative. But it is not only women who act first and think afterwards, although perhaps they confess their folly more frankly than men do. To use a homely old proverb, they put all their eggs into one basket, and, alas, not always a sound one! But "charities" are quite as uncertain as investments, and it would be very well if people took the trouble to look into them. Instead of handing over a subscription to some romantic undertaking, of the actual management of which they know nothing, they would do well to ask advice beforehand, and not after they have patronised it. The Charity Organisation is looked upon by many people as a kind of impersonal and niggardly almoner, but it is really more of an encyclopædia, which might advantageously be consulted before donations to new "missions" or unknown institutions are decided on.

### AN HONOURED GUEST.

Miss Darche made a very thorough inspection of many nurse training schools during her recent visit to England, and she is now settled back to the work which she has brought to such perfection at the New York City Training School at Blackwell's Island. Of all she accomplished here one special event appears to have transcended all others, as Miss Darche naturally looks back upon her visit to Miss Nightingale as the crowning point of her English tour, and one well worth taking a much longer voyage to accomplish. Miss Darche won such golden opinions from all those who were brought into contact with her that it is agreeable to find she considers that she received as much pleasure as she gave

### NURSING SISTERS IN INDIA.

"But it is such good pay," says some one reading through the regulations of the Indian Nursing Service, "it would be so nice to have a chance of saving money." But living in India is very costly indeed, and walking being often out of the question, hiring a conveyance becomes necessary if a Nursing Sister desires to leave her hospital quarters for even an hour or two. The charges for vehicles being so high, occasionally two ladies purchase one of their own, but the pay of a native groom and the cost of forage, &c., make the arrangement hardly economical. Then, again, in addition to boarding themselves, the Sisters combine in providing certain furniture to make their sitting-room home-like. When one lady is promoted to another station her successor is sometimes glad to buy from her the curtains, easy chairs, &c., which are on the spot. These must all be reckoned upon as expenses attending life in the Indian Empire. The allowance made by Government for outfit by no means covers the cost of the clothes which most women require to take out, but nurses are permitted to draw on their pay beforehand for this purpose. The five years' agreement is perfectly fair, as Government pays all travelling expenses, but if the climate does not agree with a nurse, and she desires to return after one hot season, she must be considerably out of pocket, and is lucky if she has private means sufficient to buy her discharge.

### GRATEFUL ACKNOWLEDGMENT.

Those interested in the Cottage Hospital at Northallerton have reason to be proud of the position it holds in public confidence. Two particularly pleasant tributes to its usefulness were paid at the recent annual meeting. One was from Mr. Swallow, the proprietor of some "roundabouts," who gave to the hospital a whole day's takings (£6 6s. 6d.) at the time of the May Fair, in grateful acknowledgment of services rendered to one of his relatives. The North-Eastern Railway Company gave a donation of £25, in recognition of the attention and care received by a fireman who was seriously injured in the Thirsk accident.

### SHORT ITEMS.

Worthing borough was divided into fourteen districts, with a nurse in each, during the recent epidemic of enteric fever.—A committee has been appointed to provide Dungannon with a Queen's nurse. At the preliminary public meeting Miss Dunn gave an interesting account of the work of the Q.V.J.I. in Dublin.—The North-Eastern Hospital for Children in Goldsmiths' Row, Hackney Road, sounds a long way off, but it is easy of access by tram and rail, and no one who goes once will fail to repeat the visit. Strangers are so kindly welcomed that they feel well repaid for their journey.— The literal errors which creep in occasionally are very humorous. A good friend of ours, and an excellent matron, in a letter received this week writes, for instance: "We have no praying probationers in this hospital." What a storm the introduction of the letter "r" would cause amongst the probationers of the institution in question did they but know how Matron has described them in this letter?—Excellent work is done, and many serious cases are treated at the Ealing Cottage Hospital; for twenty-two years Miss Reid has sustained and increased the reputation of this institution, of which she is the valued matron. —When Mr. Marion Crawford visited the New York City Training School he gave his works in 18 volumes to the Nurses' Library.

### On the Nursing of Diseases of the Stomach.

### II.—VOMITING.

Vomiting is usually preceded by nausea, during which much saliva is poured out into the mouth, and this is swallowed and with it some air. The nausea is succeeded by retching, and finally the contents of the stomach are ejected. In order for this to take place the cardiac orifice must be open and the stomach must be compressed to drive out its contents. The saliva and air swallowed during the period of nausea assist in opening the cardiac orifice, but it is also due to the behavour of the muscles in that part of the stomach. The compression is chiefly from without, the muscles of the abdominal wall contract, the glottis is closed so that air cannot, as would usually be the case, be driven out of the lungs, but all the pressure exerted by the muscular contraction is brought to bear upon the abdominal contents, and thus the stomach is pressed upon. The walls of the stomach may also contract to a slight extent and so lessen the cavity and assist in expelling the contents. During vomiting the pylorus is generally closed, but not completely closed in all cases. Bile may make its way from the duodenum through the pylorus and be mixed with the vomit, giving rise to bilious vomiting. Thus vomiting is the reverse of the normal mechanism; the contents of the stomach, instead of being driven onwards through the pylorus into the duodenum, are returned through the cardiac orifice.

Vomiting is governed by [a nervous mechanism. Nerves carry the messages to the muscles of the abdominal wall to tell them to contract, and nerves carry the messages to the muscles around the cardiac orifice, if that orifice is to be opened. Messages are sent out from a certain part of the brain, which is called the "vomiting centre." The vomiting centre receives messages carried along the nerves either from the stomach or other parts of the body, or it receives messages from other parts of the brain which make it send out the messages needed to induce vomiting. For example, food that is wrong either as regards quality or quantity is eaten; the stomach is unable to get rid of it in the usual way, and has to get rid of it by ejecting it, and in so doing uses the mechanism described. The undigested food irritates the nerves in the walls of the stomach, and a message is sent along the nerves stirring up the vomiting centre into activity, with the result that vomiting is induced, and the stomach gets rid of the offending contents.

This leads to the consideration of different forms of vomiting which are observed clinically, the first set of cases being those in which vomiting is induced by irritation of the stomach. This may be due primarily either to the presence of undigested food, as already mentioned, or poisons, such as arsenic, salts of zinc or copper, or it may be caused by alcoholic excess. These substances within the stomach give rise to inflammation of the mucous membrane; but inflammation may arise apart from substances introduced into the stomach, as the result of cold, influenza, gout, ulceration, either simple or cancerous, and the inflammation may cause vomiting. There are two classes of emetics—the direct emetics, which act upon the stomach itself; and the indirect, which act on the vomiting centre. Vomiting produced by the direct emetics comes under the form of vomiting now being considered. Indeed, some of the substances mentioned as irritant poisons are used as emetics; for example, 10-30 grs. of zinc sulphate, or 5-10 grs. of copper sulphate. Other direct emetics are warm water, salt and water, mustard and water, infusion of chamomile, ammonium carbonate (gr. 30), and alum (a teaspoonful with syrup).

The second form of vomiting is that in which there is some obstruction in some part of the alimentary canal. The immediate cause of the vomiting may be the irritation of the nerves of the alimentary canal, but it is convenient clinically to group together cases of obstructive vomiting. The obstruction may be at the pylorus, in which case vomiting may only occur at intervals of two or three days, and very large quantities of fluid are brought up. The vomited matter is generally thin, and is covered by a frothy scum. And here, it may be remarked, it is always important to note the characters of the vomit, and the nurse has the best opportunity of doing this. Points to be noticed are the quantities vomited at one time, the colour, the smell, the presence of undigested food, whether fermented or not, as indicated by the frothiness. The obstruction may be in any part of the intestine, and the causes are various for example strangulated hernia, intussusception, growth in the wall of the canal, masses of fæces blocking the canal, &c. Pain, constipation, and collapse or gradual exhaustion occur in these cases as well as vomiting, and the vomit becomes stercoraceous—that is to say, it contains matter which is on its way to become fæces, and has the odour of fæcal water.

The third form of vomiting is that due to irritation of nerves other than those of the stomach. A common example of this is tickling the fauces to make a person sick. Stimulation of the nerve of smell by some unpleasant odour causes vomiting in some persons, or to see something disgusting or to hear something disagreeable may cause a feeling of sickness. Irritation of the uterine nerves in pregnancy causes vomiting. Severe pain causes vomiting; for example, the passage of a gall stone or a stone from the kidney.

The last form of vomiting to be considered is that in which the vomiting centre is excited directly or by changes in other parts of the brain. Vomiting is a symptom in many cases of brain disease, as in tumour or abscess of the brain, and in meningitis or inflammation of the membranes covering the brain, and in apoplexy. In kidney disease vomiting often occurs, and is probably due to the condition of the blood circulating in the brain. Vomiting may be caused simply by over fatigue, and is common in sick headache or megrim. In hysteria, in some cases vomiting is a prominent symptom, and may be frequent and continue for a long time, but it is not accompanied by loss of flesh to the extent that would be expected from the frequency of the vomiting. The indirect emetics which act on the vomiting centre must here be mentioned; they are ipecacuanha, antimony, and apomorphine. The first two act on the stomach as well. Since apomorphine acts on the centre and so produces vomiting, it can be injected subcutaneously. It is very certain in its action, and its great advantage is that it can be given to patients who are unable to swallow.

Sea-sickness, and the sickness that occurs when an anæsthetic is given, have not yet been mentioned, though both are of common occurrence. They both seem to be due to nervous disturbance. When chloroform or ether is given, vomiting is much more likely to occur if food has recently been taken; hence the importance of seeing that a patient has no food for some hours before an operation is performed.

Vomiting also occurs in other diseases in which it is difficult to say if it is due to excitation of the centre directly or reflexly through other organs. Thus in scarlet fever in children vomiting is often the first symptom, and it occurs in the early stages of small-pox and cholera. Again, in the early stage of phthisis it is a very common symptom, and in "Addison's disease," vomiting with great weakness and a dark hue of the skin are prominent symptoms, but this latter is a rare disease.

### Death in Our Ranks.

The staff of the Royal Berks Hospital have sustained a severe loss in the death of one of their physicians, Dr. Moody-Ward, from septic pneumonia, on the 9th inst. He will be especially missed by the nurses and probationers, in whose welfare he was always deeply interested. He was foremost in organising regular instruction for them, and was ever ready to give them information and to help them to carry out their duties intelligently. Whilst exacting good work he won their regard and respect, and was truly "the beloved physician." His death may be regarded as a loss to the whole nursing world. He had recently been arranging papers on nursing subjects for The Hospital, and the excellence that characterised his other work guaranteed that these would prove valuable contributions.

### Women's Work.

### FEMALE MEDICAL AID FOR THE WOMEN OF INDIA.

By Mrs. Scharlieb, M.D.

(*Read at the Women's Conference at Leeds.*)

### I.—THE WOMEN OF INDIA.

It is very difficult for women born and brought up in England to realise in any degree the conditions of life which environ the women of India. Some hazy ideal vision arises of "India's coral strand"—a dream more or less beautiful, in which appear feathery palms, skies of the deepest blue, floods of golden sunshine, and gorgeous flowers.

In this romantic setting we picture the gentle Hindu girl, with her slender frame, delicate limbs, and dark, pathetic eyes. . . . The whole picture is beautiful, unfamiliar, and bewitching. It is not untruthful, however exaggerated, but it is delusive, for it expresses only one fragment, one phase of the life it professes to represent.

The life of a Hindu woman is very essentially a life of suffering, of narrowness and incompleteness. Even in infancy her troubles exist. It is but seldom that the little girl is welcome. The great desire of parents is for boys. . . . I knew a native lady who had six little girls; when the seventh was born she asked whether it were a boy, and on the question being answered in the negative, she remarked, "Then I shall never face my friends again." She turned her face to the wall and refused to be comforted. She fretted continually, took no food, and finally died when her baby was a week old.

Probably the happiest years of a Hindu girl's life are those of early childhood. . . . One day resembles another, except when some feast or religious ceremony breaks the monotony, and too soon the days of childhood are past. The little maiden is betrothed. In Brahmin families this ceremony may be performed in absolute infancy, but more generally about the age of eight or nine. . . . The ceremonies are prolonged and most wearisome, but, luckily, when they are over the child-wife remains in her father's house among familiar faces, and in many instances petted and beloved by the parents to whom her birth brought so little joy. After a time another ceremony is performed, and then the child has to leave her home and family and must go with her unknown husband to his father's house.

The severance of natural ties is total, the girl is entirely cut off from her own clan, and is henceforth reckoned in that of her husband. . . The child-wife is seldom welcome to her mother-in-law, who treats her as her servant, and exacts careful service at her hands. The poor little girl who has grown up without training or discipline now finds that she has to perform all sorts of domestic duties under the eyes of an exacting task-mistress. If she does well she is not praised, but if she does ill she is overwhelmed with a torrent of abuse, and is too often severely beaten.

Of course her husband is no comfort to her. It is against the custom for him to talk to his wife, and she dare not address him. . . . The wife is her husband's chief servant. When he comes home from school (nearly all the newly married husbands are school boys) or from work she attends him, pours water over his tired feet, lays ready the simple garments worn at home, prepares his bath, cooks his food, serves it, . . . and stands behind him while he eats. When he has eaten, what remains is for his wife.

She would be shocked indeed at any suggestion that she should share his meal or that he should wait on her. The isolated position of the child-wife is to our minds her greatest misfortune. Anyone will gladly work for those they love; no duty is menial that is done for love, but the child-wife has no loving care from her mother-in-law. no companionship with her youthful lord. . . . In her moments of leisure her only idea of relaxation is to sit on the floor chewing betel and listening to the idle stories imported from the bazaar, spiteful gossip about relations and neighbours or the abominable legends of the Hindu gods and goddesses. . . . This picture of "wasted lives and marred," of moral ruin, is sad enough, but there is a still darker page in the life of a Hindu woman, and that is widowhood. The life of a Hindu wife seems dull enough and unhappy enough to us, but what widowhood is to her we can hardly understand. It is the end of all things that made her life of value to her; with the death of her lord all her natural duties cease and all pride of position is annihilated. After a certain time has elapsed the widow, whether old or young, is led out clothed in her most costly garments, and decked with all her jewels. The procession halts at the temple, the officiating priest meets it; and with various religious rites she is divested of her rich robes, her jewels are torn from her, her head is shaved, and with shame and sorrow she enwraps herself in a coarse unbleached cloth, henceforth her only garment . . . . Strange to say she is an object of scorn and contempt, her name is a byeword and reproach, her very touch is pollution.

Such is the life of a Hindu woman—a neglected child, a servitor's wife, and finally a death in life scarcely more merciful than the funeral pyre from which the strong arm of the British Government alone saves her.

(*To be continued.*)

### Nursing in the United States.

(By Miss Darche, Lady Superintendent New York Training School for Nurses, Blackwall Island.)

### PROPER ORGANISATION OF TRAINING SCHOOLS.

(*Continued.*)

The Nightingale School must ever stand alone as unique in its scope and organisation; the pioneer school of all schools, the conception of a noble woman whose generosity and philanthropy set in motion a system of caring for the sick, which has brought light and comfort into more dark places than, perhaps, any other movement of this century.

But to return to our subject. We now approach the third phase of training-school organisation as seen from an American standpoint, and this is the School Registry, managed in the interests of its graduate nurses. Our first board of lady managers having provided a reformed system of nursing in the wards of the great hospital, as they had set out to do, and having also in the process developed a nurse-training school for women, now decided to carry on their good work a little further, and institute a plan of registration which would enable their graduate nurses to get employment readily under the auspices of the school, and would at the same time guarantee protection to those seeking the services of a nurse. The plan adopted was a very simple one. The graduate nurse engaged a room in some part of the city to which she could resort when off duty, with the understanding that when available for duty she would report to the registry, and hold herself in readiness to answer any call that should be sent to her. The public generally, and physicians in particular, were notified that duly trained and qualified nurses could be secured through the school registry. A reasonable weekly fee was decided upon, which the nurses were allowed to charge for their services while nursing in private families. A few sensible rules were formulated for the purpose of protecting the nurses from unjust or an undue amount of continuous duty, suggesting also their obligations to the registry, and to the families in which they should engage to nurse. A small yearly fee, sufficient to cover the expense of registry calls, was instituted, to be paid by the nurse; the superintendent of the school was made responsible for the registry management, subject—as in the school management—to the approval of the managing committee; and the third phase of training school organisation was completed. That this plan of registration succeeded is not surprising when we consider the forces thus set in motion. The newly-graduated nurse as she entered upon private duty under the supervision of her school felt both stimulated and encouraged to prove herself worthy in this new field of labour. The school management, feeling itself thus closely and directly represented to a critical public, naturally felt desirous of promoting to a higher degree of excellence the training and education of its student-nurse. The medical profession, finding a ready means of procuring trained nurses for private cases, patronised the registry, and the registry thus established continued to be appreciated and to flourish. Thus was the school in its threefold aspect completed, and stands to-day representative of training-school organisation in America.

It is true, many and devious variations from this original design, to suit the exigencies of peculiar hospital management, have been made, but it is only when the fundamental principles first laid down by Miss Nightingale at St. Thomas's Hospital, London, and afterwards introduced here, are adhered to in all their essential entirety, that there will be harmonious and successful training-school development and organisation.

The essential elements of proper training-school organisation then may be briefly stated thus: (1) A hospital containing a fair proportion of medical, surgical, and obstetrical cases; the hospital constructed with ordinary conveniences for the care of patients. (2) The understanding that the nursing is to be considered a distinct department in itself, whether managed under the hospital or a separate board of control. (3) As head a superintendent, who will herself be a trained nurse and a woman of considerable executive ability, (4) A home for nurses removed from close proximity to the hospitals wards, and so arranged that nurses when off duty may enjoy the ordinary comforts of home life. (5) A definite course of lessons and lectures, embracing all theoretical instruction necessary in the education of a nurse. (6) A board of lecturers and examiners, composed of visiting physicians and surgeons of the hospital, and who, with the superintendent, will conduct the final examinations and award the diplomas. (7) A school registry, to be managed in the interests of the graduate nurses.

(*To be continued.*)

### Notes and Queries.

### Queries.

(220) *Certificate.*—Please tell me if a certificate gained at an asylum justifies me in passing as a trained nurse? Will it be accepted as sufficient qualification by any Nurses' Co-operation?—*E. F.*

(221) *Important Point.*—I shall be greatly obliged for information on a point which is of some importance to myself. Can I obtain any redress when a journal, in which I should certainly never advertise, inserts in its columns an advertisement copied from another publication to which I had sent it? This may sound a matter of small importance, but it is not so to me and to others who, like myself, are unaware of the legal rights in a matter calculated to injure my position with my patrons.

(222) *Guidance.*—What is a good handbook for a young mother—not too expensive?—*Gravesend.*

(223) *Porter.*—How can I obtain a situation as porter in a hospital?—*G.E.S.*

(224) *Code.*—Kindly inform me how I can obtain the code of rules for "Prevention of Infectious Diseases in Schools," passed by the Medical Officers of Schools' Association.—*H.E.M.*

(225) *Moderate.*—What is the best kind of luggage for a private nurse? —*Subscriber.*

### Answers.

(220) *Certificate* (*E. F.*).—Such a certificate makes you simply "a nurse trained in mental nursing." See paragraph in "Nursing News" this week. Unless you have also a certificate for general training you would not be eligible for the Nurses' Co-operation, 8, New Cavendish Street. Of course, there may be other associations where, the standard being lower, they might admit you.

(221)—Consult a solicitor, who will no doubt be able to promptly stop proceedings which are a fraud upon the public, and must, as in this case, prove harmful to the advertiser.

(222) *Guidance* (*Gravesend*).—"Handbook for Mothers," by Dr. Jane Walker, price 2s. 6d.

(223) *Porter* (*G. E. S.*)—You had better advertise.

(224) *Code* (*H. E. M.*)—You had better order a copy through any good bookseller.

(225) *Moderate* (*Subscriber*).—A small light trunk which one person can carry. At the Homœopathic Hospital, in Great Ormond Street, they are provided for the private nurses, but most institutions content themselves with giving general instructions on the subject.

### Nursing in Switzerland.

By Dr. Charles Kraft, Director.

### PRINCIPLES OF LA SOURCE.—II.

#### The Council.

La Source is committed to the administration and to the care of a Council composed of twenty-five members. The Council forms an association which meets twice a year to deliberate on every question pertaining to the school. In the interval between these meetings the Council delegates its powers to a committee composed of three members.

#### The Directors.

The Normal Evangelical School of Independent Nurses for the Sick was from 1859 to 1891 a purely private institution. M. and Mme. de Gasparin confided the education of the pupils to a Director, and this Director was responsible to them only. MM. Muller, Panchaud, Reymond, filled successively this office for 32 years. The latter, M. A. Reymond, pastor, had directed the school for 28 consecutive years. Let me recall here that M. Reymond was always the ardent defender of the principles of La Source, that these fifty-six generations of pupils (two classes in a year) were the favourite work of his life, and that the nurses whom he trained remain sincerely attached to him both by bonds of gratitude and of respectful affection. The school was changed in 1891. M. Reymond having retired from his arduous labour, the author of these lines, appointed by Mme. de Gasparin, became the Director of La Source. In this position he directs the progress of the school, lays down the course of study, appoints the practical work of the pupils, provides for their support, supervises the interior workings of the institution, is responsible for the preservation of all property, carries out the wishes of the Council, &c. The Director chooses his own assistants and is responsible for them. The present Director—a physician—has established a private clinic in the school building which is under his direct care. In this clinic the pupils obtain a large proportion of their practical work. Dr. Kraft is assisted in part of his work by Mdlle. Marie Le Coultre. She has the title of Directress, and has the immediate charge of the establishment; she regulates the domestic arrangements of the school and clinic, assigns various tasks to the pupils, hears recitations, and gives special attention to their training in household affairs, besides being charged with a part of their moral teaching. The Directress lives in the school building. The Director makes biannual reports to the Council. He edits the journal of the school. This journal is called *La Source*, and appears every three months.

#### The Location.

La Source, a beautiful villa surrounded by gardens, is situated in the near neighbourhood of Lausanne. The air is pure. In front of the building stretch the shores and blue waters of Lake Leman, while beyond is unfolded the vast panorama of the Alps of Savoy and the Jura. The property comprises a principal building with adjuncts, in all twenty buildings. Eleven of them are occupied by the offices of the Director, the rooms of the Directress and pupils, and nine by the clinic. There is running water through all parts of the buildings, and there are bathing accommodations, operation-rooms, isolated rooms for contagious diseases, &c. In short, nothing is lacking which is necessary for the recovery and the comfort of the nurses and patients.

#### Instruction.

The pupils of La Source are taught practically and theoretically. The theoretical course covers about 120 hours, distributed an hour a day over the five months of training. The complete course is divided into five branches—hygiene, anatomy, physiology, pathology, and therapeutics. These subjects are taken up in succession, each for one month. This course, given by the Director, comprises obstetrics to the extent necessary for the care of women in child-bed. La Source does not furnish a complete course of midwifery. Examinations: After the five months' course of teaching the pupils, both internes and externes, undergo an oral examination upon the five branches taught. The markings which they obtain, combined with the markings for practical work given by the Director and the Directress, determines the grade of their diploma, "very satisfactory," or "satisfactory," or "sufficient." The practical work is obtained: (1) Among the sick poor of the town, who are for the most part placed under the care of the Director, and are visited by the pupils; and (2) with the patients on the Director's private clinic and polyclinic.

Head Nurse of the Clinic.

The best ones among the pupils may prolong their service. They then take the title, and assume the duties of the chief of the clinic, exercising a direct supervision over the young pupils working in the town or in the clinic. They keep order, take care of the dressings, instruments, and appliances used by the Director in operations, and thus perfect themselves further in the practice of their art. Each year sixteen pupils, internes, without counting a smaller but variable number of externes, enter La Source and leave it furnished with diplomas or without. La Source has always at least four internes, at most eight, receiving their training there.

### Presentations.

Kent Nursing Institution.—At the monthly meeting of the committee on the 8th inst., the Dowager Countess of Aylesford, on behalf of the committee, presented to the Lady Superintendent (Miss Helen Ligertwood) a silver-gilt medal, as a slight recognition of her valuable services to the institution during the past eight years.

### Prince Alfred Hospital, Sydney.

### THE NURSES' DINING-ROOM.

The "general" or "nurses" dining-room at the new Home connected with the Prince Alfred Hospital, Sydney, is a very fine hall 42 feet by 29 feet, which opens on to a verandah. It is suitably and prettily furnished, and, after the pleasant fashion which the Prince of Wales has adopted at Sandringham, the meals are served at separate tables. There is something very agreeable and homelike in a custom which permits nurses to form sociable little parties in this way, and the practice might well be recommended to all institutions in England with a moderate staff of nurses and an adequate supply of servants. A lift from the kitchen brings the food conveniently near to the dining-room at the Prince Alfred, and on the same level as the latter is the excellent dressing-room, where hot and cold water and a liberal allowance of basins, looking-glasses, &c., enable the nurses, however busy, to "get ready for dinner" with a minimum of trouble and fatigue. The doors and skirting boards throughout the Home are of beautifully polished cedar wood, and nothing has been spared to make the rest of the building, like the fine dining-room shown in the illustration, a model to all institutions.

Prince Alfred Hospital, Sydney—The Nurses' Dining-Room.

### Wants and Workers.

The Honorary Secretary (Mr. E. P. Smith), North Kensington Friendly Workers Among the Poor, 28, Colville Square, Notting Hill, W., writes: I know of a woman, who bears an excellent character, who wants a pair of deformity boots for her son. The Surgical Aid Society require six letters for the boots. At present I have none myself. I shall be much obliged if you will help me by sending or asking some one to send me some Surgical Aid letters.

### Our American Letter.

There is always some one thing specially before members of the nursing profession. It varies in kind and in degree doubtless, but yet for a time it engrosses our attention.

For a large subject just now we have prominently before us a thing which, although beneath contempt, demands attention, and it also requires to be put down with a strong hand. This is the dangerous attempt to persuade folks that they can become trained nurses by correspondence. This system is at present advertised by more than one agency.

It is bad enough to find women posing as trained nurses after twelve months' hospital experience, but for them to pass themselves off on the public as such, after a correspondence course, supplemented by "a little experience," is worse.

Our admirable nursing organ, *The Trained Nurse*, does good service by exposing in this month's journal the names of the advocates of this plan, and also the address of a person who advertises for "the names of those who desire to become trained nurses by studying at home."

It will be well if American aspirants read *The Trained Nurse* before responding to this seductive invitation; they might, as the papers say, "hear of something to their advantage," or, at any rate, "to their enlightenment."

On All Saints' Day Miss Rodman was set apart for the office and work of a deaconess in St. Stephen's Church, Philadelphia. She is a graduate of the Church Training and Deaconess House in Spencer Street. The training in nursing consists of two or three months passed in one of the city training schools, and apparently the amount of instruction given is similar to the preparation of English Church Army nurses. But over here we wisely avoid misplacing the title "nurse." There is now a gynæcological ward at the Boston City Hospital which is already proving a valuable adjunct to a most useful hospital. Twenty-five nurses have graduated at the training school during the past year, and a larger nurses' home is already required for the staff at the City Hospital.

The fine building of the Royal Victoria Hospital at Montreal is completed, and Miss Draper is working up the supplies of linen and hospital furnishing ready for its occupation, and after her previous hard work at Cook's County Hospital she finds her present occupation a refreshment.

### Everybody's Opinion.

[Correspondence on all subjects is invited, but we cannot in any way be responsible for the opinions expressed by our correspondents. No communications can be entertained if the name and address of the correspondent is not given, or unless one side of the paper only be written on.]

### "THE NEW ORDER OF PRACTITIONERS"—ASK THE DOCTOR?

By a District Nurse in the Country.

A "Six Years' Country District Nurse" writes: Will you allow me to make a few remarks regarding a notice which appeared in the "Nursing Mirror" under the headline "Ask the Doctor." No doubt the letters of the Queen's Nurses were far more orthodox than the advice of the nurse of the North London Nursing Association, but orthodoxy on these points is not always any more than on many others the most practical thing. Putting aside the fact that even in London, with its large number of free dispensaries and out-patient departments of hospitals, patients with ulcers of leg are not those who receive much attention from medical men; in the country the overworked parish doctor is in most cases the only person to give medical aid to the poorer classes. Having worked in the country, as I believe does the nurse for whose benefit the unorthodox advice was given, I can speak from personal experience, and I know that the advice in question is sound and good. A country district nurse at any rate must be able to treat ulcers of leg, as well as any other things, without actual help from the doctor, although nominally the patient may be under the care of the latter. I cannot help adding what a country practitioner—a F.R.C.S.—said to me a short time ago. The fault (he said) he had to find with most trained nurses was that they could only carry out his literal orders; they could not act on their own responsibility, or have an intelligent opinion on a case.

By the Superintendent North London Nursing Association.

"S. de Luttichau, Superintendent, N.L.N.A.," writes: Having seen a notice in the "Nursing Mirror" commenting on a letter written by a nurse of the North London Nursing Association, I must ask you to be kind enough to insert the following explanation of the letter in question. It was called forth by an appeal from "Nurse Marian" that some fellow nurse would advise her as to the best means of deodorising foul ulcers of leg. Nurse Marian stated that she had in vain sought advice from doctor and chemist. The nurse of the North London Nursing Association, who is, I need hardly say, a fully trained hospital nurse, and who has besides an exceptionally long experience in district work, having given instrvctions on the subject which even a F.R.C.S. is bound to allow as good, ends up with what perhaps sounds extraordinary advice: "That a nurse should not seek advice from doctors or chemists in the management of ulcers of leg, but should learn her own duty on the subject in a good District Home." I can only say that, as a rule, doctors will not be troubled to give advice on the management of ulcers of leg. Unless, therefore, our nurses were as they are, so well-trained that they can as a rule manage such cases without medical aid, the numerous sufferers from that painful complaint would, I am afraid, have to be content to continue to have their ulcers unhealed and foul. In these cases it is quite necessary that the person who dresses them, *i.e.*, the nurse, should thoroughly understand their local treatment, and be able to vary it as required. No doctor would see the case often enough for that, and my own experience of many years as a superintendent goes to prove that practitioners expect a nurse to know her work in this way.

By the Author of the Statement.

"A District Nurse" writes: As the writer of the advice in "Nursing Notes" for October, *re* a foul ulcer of leg, allow me to explain the circumstances which are misunderstood. An inexperienced nurse wrote from the country to ask what could be done, in the way of dressing, to keep such an ulcer inoffensive. As a trained district nurse of long experience I replied by giving her a description of the mode of application of two dressings of well-known and proved value. In conclusion, I suggested that instead of asking doctors and chemists—as she represented herself as doing—for help in the matter, she should get training as a district nurse. Surely it is better a nurse should know what she is about independently of the doctor, and so if he does give instructions she is able to carry them out effectually. "F.R.C.S.," who criticised my remarks, was mistaken in thinking that the advice I gave made any pretence to be a secret cure. Nothing I said could be fairly so interpreted. Again, in saying an ulcer is not a wound I made a technical mistake, for which I am sorry. But of course an ulcer is correctly described as a sore, which it would, I think, generally be called. I trust that in common fairness you will insert this letter in your Nursing Supplement.

### The Muses' Looking Glass.

### A HOSPITAL "SING."

Just as the charity dinner is a distinctly metropolitan institution, the hospital "sing" is an essentially provincial one. It probably originated with a few working men, with vocal or instrumental skill, who desire to benefit the hospital at the same time that they make an occasion for the exercise of their voices or violins. The hospital takes no part in the management of the affair; its share is confined to the pleasant duty of receiving the money raised by its voluntary friends. The amateur musicians form themselves into a committee, with chairman, secretary, and treasurer, and, when the day's work is over, meet at some room lent gratuitously for the purpose, fix the day for the "sing," and select the tunes. All that is now required is fine weather—local enthusiasm does the rest.

A Sunday is chosen for the event; and if on that particular Sunday afternoon pleasure or duty impels you in the direction of the "sing field"—for that is the name acquired by the field as year after year the singers meet in it—you will find yourself one of a gradually increasing stream, composed of democracy in its Sunday clothes.

The "sing," by its regular recurrence, has marked itself as a red-letter day in their calendar—a day to which they look forward for weeks before it arrives. Heavily-freighted waggonettes, dog-carts, and whitechapels raise a cloud of dust until they have discharged their contents, and fallen into line outside the field. All along the line of route boys offer the programme of the "sing," and for the sum of one penny you can become the possessor of an advertising cover containing, in clearly printed type, the words and music of the hymns to be sung.

At the entrances through which the people are pouring, an organized staff of collectors stands with boxes and in charge of sheets for free-will offerings. Everyone is asked to give a trifle, but there is no compulsion. Now the stand—erected gratuitously by a local joiner—is full; and the conductor with his baton takes his seat, turns the leaves of the music in front of him, beckons the orchestra and singers, rattles the music stand in orthodox fashion, and cutting the air with a wide sweep, leads off the orchestra as it plays over the melody of the first hymn. Then the singers—sopranos and altos, tenors, and basses—fill their chests, and the hymn bursts forth upon the summer breeze, to reverberate in fuller volume still as it is taken up by the thousands who are within the enclosure.

It is no mean orchestra and chorus that the conductor controls. Three hundred musicians acknowledge his sway and lead the singing. The hymn finishes amid reverent silence. There is no applause, and even the choruses from the "Messiah," admirably as they are rendered, though they elicit marks of approval, call forth no murmur of applause. The reverent spirit in which the music is received, the respect which is paid to the sacredness of the day, are quite noteworthy. It is a sacred concert and the behaviour of the crowd is in keeping with the character of the music, and seems indeed to be deepened by it.

The musicians and conductor give their services. Some of them come from a distance. For them tea is provided, and all the work is done voluntarily as a labour of love. The managers and organisers of the event are working men whose names will never be known outside the circles in which they move.

What are the results? The report of one hospital in a district famous for its "sings," acknowledges the receipt of £121 from seven of these musical festivals. This may not seem a large sum, but when it is a regularly recurring annual gift, which the hospital can always anticipate, it is practically a huge annual subscription. Moreover there is no commission to deduct for collection, whilst the labour involved in collecting that sum in guineas or multiples of a guinea is absent. When, however, it is remembered that this sum is an aggregation of coppers and small silver, the financial success of the "sings" is remarkable.

And whilst the cause of charity is brought before the people, they, in return, are brought within the refining influence of music, which should act as an antidote to ditties of the "Monte Carlo" and "Ta-ra-ra-boom-de-ay" type. To many of the toilers in mills and works, the "sing" is one of the events of the year; and they return to their tasks all the better for the break in the monotony of their lives, for the afternoon's revel in the sunshine and fresh air, uncontaminated as they are by any temptation more dangerous than flirtation and sweethearting.

### Where to Go.

### PICTURES.

Saturday was a day of "private views"—the most interesting of which was of Mr. Arthur Tomson's cat pictures at Mr. Van Wisselingh's Dutch Gallery. There were some fifty pictures in all, and nearly all mediums had had their share in producing them; oil and water colour and pastel. Mrs. Graham Tomson is well-known as a poetess, and as the editress of an anthology on cats, for she has a passion for all the feline race, and her pretty house and garden in St. John's Wood Road, are a veritable cat paradise, where the beautiful creatures seem to show off their supple shapes and sinuous attitudes as if in gratitude for the care and kindness they receive. Mr. Tomson, has delighted in painting them in all humours. We see them sporting on the branches (presumably after birds), at the toilette, "the wooing o't," stealing from off the dinner table, &c. All cat lovers should make a point of looking in at 14, Brook Street (two doors from Bond Street), where Mr. Van Wisselingh makes all comers welcome, without the usually shilling "sesame." Mr. Van Wisselingh is a very rich man and a collector of great discrimination and experience. He can afford to keep his best pictures for his own enjoyment at the beautiful house he owns at Pinner, to visit which is an even greater treat than to inspect the Brook Street galleries. His family have been well known in Amsterdam for generations, and it is only on his charming wife's account, who is an Englishwoman, with whom the Dutch climate disagrees, that he has started a branch establishment in London, and imported his exquisite old Dutch furniture, priceless heirlooms, family portraits, and innumerable rare books to the nest he has formed for her beyond London fogs at rural Pinner. His private collection of Corots, Rousseaus, Daubignys, Maris, Manets, Whistlers, &c., are things to dream of, for such exquisite examples of these masters are rarely to be found. There is, however, a beautiful Corot now for sale at the Dutch Gallery which connoisseurs consider cheap at £2,000. The private view was brilliant in spite of gloomy weather. Mrs. Tomson, who was much admired by Rossetti, whose ladies she much resembles, looked extremely well, and was picturesquely dressed. Aubrey Beardsley, the youthful *décadent* whose illustrations to Oscar Wilde's "Salomé" have taken everybody's breath away, was chaperoning his pretty sister, the charming Anglo-Saxon scholar. Amidst the throng of picture cats and interested beholders something wonderful in raiment stood out prominently. This was a bodice worn by one lady which resembled closely a scale armour of green beetles' wings. The wearer further showed her courage by donning a velvet coal scuttle bonnet with the towering plumes of our grandmothers' time. Mr. Tomson's brother-in-law, Wilfred Ball, was also holding a private view at Agnew's. The pictures are all the same size, all framed alike, hung two and two all round the gallery, and the subjects and treatment are as monotonous as the size and the frames. They are supposed to represent Egypt and the Nile, and for the purposes of a Christmas card are quite adequate. We felt inclined to turn them round to ascertain if the mottos were on the backs and to inquire of the attendant what their price was by the dozen.

### The Book World for Women and Nurses.

[We invite Correspondence, Criticism, Enquiries, and Notes on Books likely to interest Women and Nurses. Address, Editor, The Hospital (Nurses' Book World), 428, Strand, W.C.]

Adventures in Mashonaland. By Two Hospital Nurses. (London: Macmillan and Co. 1893.)

The saving clause, so to speak, in the title of this work exists in its latter half. One feels, that having been already through "The Dark Continent" under the guidance of Livingstone, Paul du Challu, and Mr. Stanley, one has had, perhaps, a sufficiency of mental excursions in the same direction; but under a female pioneer the case assumes a fresh aspect. And as one turns the pages of this volume the interest awakened in its title sustains itself in no ordinary degree. Miss Rose Blennerhasset and Miss Lucy Sleeman, the two "Hospital Nurses" are no ordinary women; indeed, they show in their quietly-sustained powers of endurance the courage which a woman's nature is capable of, but which, from force of circumstances, is not always brought out. It was brought out anyhow in the case of these two; and Sister Aimée, as she is called, tells her story in an unaffected manner, which merits much praise. It was in the spring of 1891 when these two young women heard that Dr. Knight Bruce was anxious to take some nurses up to his new diocese at Fort Salisbury, where he projected establishing several hospitals, for which purpose they volunteered their services. At this time they were in Africa, having gone out for "change of work" to nurse an epidemic of typhoid then rife among the inhabitants of Johannisburg. Just then the Chartered Company's Expedition to Mashonaland was in everyone's mouth. The Bishop of Bloemfontein had given up his former diocese for that of Mashonaland, and considerable interest was aroused. But the plans of the mission had not assumed any definite shape, and with the Portuguese question coming to a crisis, and the unsettled state of the country, this was readily understood. On all sides the two nurses were advised to give up the scheme, but they were not to be daunted. Meanwhile the Bishop had preceded them up country towards the seat of the future mission, and as best they could under the difficulties which arose at every step, Sister Aimée and her companion followed. But there were some things beyond difficulties with which the two had to contend; dangers, in forms as varied as they were many, menaced the path which they pursued. To get to Fort Salisbury, the field of their future labours, the route to be traversed was strewn with many thorns. Firstly, there were several days to be undergone steaming in a coasting steamer up the Pûngué, an almost unexplored river, along whose marshy banks crowded swarms of crocodiles. The heat was intense, there was no awning, whilst at night thick white mists enshrouded the land. Life was not all *couleur de rose*, Sister Aimée writes, during that time. Indeed, the descriptions of this time read like a bad nightmare. But the chief interest in the book centres itself round that part of the journey which the nurses at length performed on foot. This expedition has had no precedent in the annals of womankind, but though they knew this they were not to be deterred from venturing. Perhaps they had not quite reckoned the cost before they started, but anyhow one reads they set out. It was

**THE BOOK WORLD FOR WOMEN AND NURSES**—*continued.*

a task needing no little endurance, however, on their part. No other means of travelling presented itself, and so taking their lives into their own hands, they commenced their arduous journey. The road—if road it could be called—for as yet it was unmade, led them for miles through marshy swamps, where the grass grew high above their waists. Through bogs, over which the native escorts carried them; then through long stretches of parched-up ground where no water was. One follows Sister Aimée's account of this adventure with bated breath, and one asks how she survived to tell the tale; but with the exception of losing all luggage and tramping bootless, no very serious evil was resultant on this walk in the wilds. And after many days their goal was reached, but the interest of the "adventures" does not cease even here. The Bishop went back to England at this point to collect "funds for his work," and Sister Aimée and Sister Lucy Sleeman was left in charge of the "hospital," as it was called. And here fresh dangers awaited the two nurses. One reads of a tent being burnt down over a patient's head! of a leopard looking in at a door when another lay delirious in a fever, and so on, for it is hard among so many incidents to particularise any special one. And it is not only of horrors that we are told throughout the book; it has its humorous as indeed its instructive side. Under Miss Blennerhassett's guidance we get a clear insight into the lives and manners of the natives amongst whom she was thrown. A certain interest, too, centres round the character of "Old Wilkins," who had long ago served under Livingstone, which fact the old man constantly brings forward. "Should a man who has been with Livingstone be ordered about?" he asks, on convenient occasions. The great drawback to the book is an absence of illustrations; a few of these would have helped more completely to impress on our minds the scenes the writer's pen describes. One feels this all through; but yet one cannot say that the work is spoilt for want of drawings, but it would have been much enhanced by their presence. In other ways the book is well got up, and of an attractive form. Not daunted by her experiences as a nurse in Africa, we learn now that Sister Aimée is shortly to be ensconced as Matron to the Civil Hospital at St. Helena.

Six Common Things. By E. F. Benson. (London: Osgood, McIlvaine, and Co.)

Mr. Benson has taught us in this little volume to look into life from the standpoint of its pathos. Under the title of "Six Common Things," the author has published a series of short tales, chiefly concerning incidents with which most of us can probably find a sequence in our own lives if we choose to reflect, but which most of us also have passed by, nor cared to see. Mr. Benson, undoubtedly, has great powers of observation, and what is perhaps somewhat rarer, he possesses the gift of conveying to others what he has seen. These stories, each of them, for it is immaterial to specialise any one, are certainly well calculated to fulfil the end the writer has in view, and if alone from the supremely artistic manner in which they are written, will assuredly awaken our sympathy and our interests in the every-day life of our fellow creature. Perhaps the spirit in which Mr. Benson evolved this, his latest publication, is best translated in his own words: "The pathos," he says, "of small and trivial disappointments has to me a heart-searching sadness which I feel to be quite unreasonable, but against which I am perfectly powerless. The great tragic figures of history have a certain recompense. . . . Simply because they are mighty we feel the keenness of them less; and it is in the small unnoticed sorrows of average people that I realise most deeply the infinite pathos of human life."

## Figures and Tables

**Figure f1:**